# Radiological Findings in Young Children Investigated for Tuberculosis in Mozambique

**DOI:** 10.1371/journal.pone.0127323

**Published:** 2015-05-28

**Authors:** Alberto L. García-Basteiro, Elisa López-Varela, Orvalho Joaquim Augusto, Kizito Gondo, José Muñoz, Jahit Sacarlal, Ben Marais, Pedro L. Alonso, José L. Ribó

**Affiliations:** 1 Centro de Investigação em Saude de Manhiça (CISM). Rua 12, Cambeve CP 1929 Maputo, Mozambique; 2 ISGlobal, Barcelona Ctr. Int. Health Res. (CRESIB), Hospital Clínic—Universitat de Barcelona, Rossello, 132, 08036, Barcelona, Spain; 3 Marie Bashir Institute for Infectious Diseases and Biosecurity Institute (MBI) and The Children’s Hospital at Westmead, The University of Sydney, Australia. Westmead NSW 2145 Australia; 4 Radiology Department, Hospital San Juan de Dios. Passeig Sant Joan de Déu, 2. 08950 Esplugues de Llobregat, Barcelona, Spain; Fundació Institut d’Investigació en Ciències de la Salut Germans Trias i Pujol. Universitat Autònoma de Barcelona. CIBERES, SPAIN

## Abstract

**Introduction:**

Chest radiography remains a critical tool for diagnosing intrathoracic tuberculosis (TB) in young children who are unable to expectorate. We describe the radiological findings in children under 3 years of age investigated for TB in the district of Manhiça, southern Mozambique, an area with a high prevalence of TB and HIV.

**Methods:**

Digital antero-posterior and lateral projections were performed and reviewed by two independent readers, using a standardized template. Readers included a local pediatrician and a pediatric radiologist blinded to all clinical information. International consensus case definitions for intra-thoracic TB in children were applied.

**Results:**

A total of 766 children were evaluated of whom 43 (5.6%) had TB. The most frequent lesion found in TB cases was air space consolidation (65.1%), followed by suggestive hilar lymphadenopathy (17.1%) and pleural effusion (7.0%). Air space consolidation was significantly more common in TB cases than in non-TB cases (odds ratio 8.9; 95% CI: 1.6-50.5), as were hilar lymphadenopathy (OR 17.2; 95% CI: 5.7-52.1). The only case with miliary infiltrates and 3 with pleural effusions occurred in HIV-infected children.

**Conclusion:**

Frequent air space consolidation complicates radiological distinction between TB and bacterial pneumonia in young children, underscoring the need for epidemiological contextualization and consideration of all relevant signs and symptoms.

## Introduction

Childhood tuberculosis (TB) is a leading cause of respiratory disease in TB endemic areas. The World Health Organization (WHO) estimates that 550 000 children developed TB in 2013[1], but recent modeling studies suggest that the burden could be much higher [2,3]. Young children, and immunocompromised individuals, have an increased risk of developing active disease following *M*. *tuberculosis* infection [4].

The diagnosis of TB is particularly challenging in young children, given the non-specific nature of their symptoms, difficulties in obtaining samples for microbiological examination and the often pauci-bacillary nature of their disease [4]. Liquid culture, the accepted diagnostic reference standard, is positive in less than 50% of children treated for TB [5,6], although this varies depending on the degree of lung involvement [7]. Moreover, liquid culture or molecular diagnostic tests are not available in many low resource-limited settings[8]. In everyday practice, TB diagnosis in young children relies heavily on exposure to an infectious source case or immunological evidence of *M*. *tuberculosis* infection, together with findings suggestive of tuberculosis (TB) on the chest radiograph (CXR).

CXR remains a critical tool for diagnosing intrathoracic TB which is the most common presentation of TB in children [9]. In fact, CXR signs suggestive of TB are considered essential to establish a diagnosis of probable intrathoracic TB, according to international consensus clinical case definitions [10]. The most common radiological finding associated with TB in children is perihilar or mediastinal lymphadenopathy [11,12]. Cavitary lesions are rare [12], except in very young infants and HIV infected children [13], or with the emergence of adult-type disease during adolescence.

Few studies have described CXR findings in young children evaluated for TB, comparing TB cases with those considered not to have TB. We describe radiological findings in children under 3 years of age investigated for TB in Mozambique, in an area endemic for both TB and HIV[14]

## Methods

A prospective descriptive study of young children (<3 years of age) evaluated for TB was conducted at the Manhiça Health Research Center (CISM), located in Southern Mozambique, over a 1-year period (October 2011–2012).[15] Socio-demographic characteristics of the population living in the district of Manhiça have been described in detail elsewhere [16]. In 2011, the incidence rate of confirmed TB among HIV infected adults aged 18–47 was 847 per 100.000 [14]. The overall estimated TB incidence rate in Mozambique is 552 per 100.000 population [17]. Most children in the country receive Bacille Calmette-Guerin (BCG) vaccination at birth; coverage is around 90% [18].

Children with symptoms suspicious of TB and those in close contact with a sputum smear-positive TB case were evaluated. Symptoms suspicious of TB included: cough for ≥14 days not responding to a course of antibiotics; referred fever for ≥14 days after common causes like malaria were excluded; weight loss/failure to thrive(defined as under 60% weight for height, failure to gain weight for >2 months, any loss of weight unresponsive to nutritional rehabilitation). Full details on inclusion criteria are described elsewhere.[15]

Evaluation included a physical examination, HIV rapid antibody test (Determine, Abbott Laboratories), tuberculin skin test (TST) and CXR. A positive TST was defined as an induration of ≥5mm for HIV-infected or malnourished children and ≥10mm for the rest of participants. HIV infection was defined as a positive antibody test in children >18 months (Determine, Abbott Laboratories and confirmed with Unigold, Trinity Biotech); or positive HIV PCR in those <18 months; or a strong clinical suspicion with positive antibody test in the absence of a PCR result.

One induced sputum (IS) and one gastric aspirate (GA) were collected from each participant and evaluated by smear microscopy and culture. All participants included had at least one follow up visit arranged within six months of recruitment regardless of initial disease classification, to assess symptom resolution with or without TB treatment. Those who remained symptomatic were re-assessed, by repeated CXR and collection of new samples. TB cases received 6-months of supervised treatment according to the National TB Control Program protocol. Non diseased-contacts were referred to the NTP for isoniazid preventive treatment (IPT) initiation.

### Chest radiograph reading and reporting

CXRs were performed with a digital X-ray machine (Philips—Optimus 50). Antero-posterior (AP) and lateral projections were performed. Chest X rays were reviewed by two observers. Initial reading was done by a local pediatrician (ELV) and second reading by an experienced pediatric radiologist in Barcelona, Spain, (JLR) who was blinded to all clinical information. For the purpose of this study, the blinded reading was used. CXRs were evaluated and reported using the CXR tool developed by Andronikou [10]. This tool (**annex 1**) first assesses the quality of the CXR and then evaluates the presence and location of airway compression and/or tracheal displacement, soft tissue density suggestive of hilar lymphadenopathy, air space opacification (consolidation), nodular images (miliary or larger; widespread and bilateral), pleural effusion, cavities, calcified parenchyma or vertebral spondylitis. If at least one of the above features is found, the CXR is classified as “consistent with tuberculosis”.

### Case definitions

We applied international consensus case definitions for intra-thoracic TB in children, which differentiates definite (microbiologically-confirmed), probable and possible TB, as well as unlikely TB and not TB [10]. All children with definite and probable TB were recognized as a “TB case”. Children with definite TB had compatible symptoms and positive *M*. *tuberculosis* culture. Probable TB cases had compatible symptoms, suggestive CXR and at least one of the following: TB exposure, positive tuberculosis skin test (TST) or positive response to TB treatment. For the purpose of this study all other children were regarded as “non TB cases”.

### Data analysis and ethics

Clinical data from participants was double entered using OpenClinica software and laboratory information was retrieved using Servolab platform. DICOM software was used to visualize and read the chest X rays. Statistical analysis was done using Stata 13.0 (StataCorp 2013, College Station, TX). The study protocol was approved by the Mozambican National Bioethics Committee and the Hospital Clinic of Barcelona Ethics Review Committee. Written informed consent was obtained from the caretakers of all study participants. The individuals whose chest X rays are shown in this manuscript have given written informed consent (as outlined in PLOS consent form) to publish these case details.

## Results

A total of 766 TB presumptive cases had at least one CXR at admission. Of them, 752 (98.1%) cases had the AP projection and 481 (62.8%) had the lateral view (467 cases had both). The technical quality of the chest X ray was generally good. At admission, 76.4% of all chest X rays were classified as acceptable (71.9% and 79.3% of AP and lateral views respectively) and only 1.9% as illegible. Around 55.1% of the participants were male and 50.8% were between 12 and 23 months of age. There were 43 Tb cases, 13 of them (30.2%) were microbiologically confirmed (7 in GA, 4 in IS and 2 both in GA and IS). The prevalence of HIV infection in children admitted into the study was 13.1% and 46.5% among TB cases. Baseline characteristics of these TB presumptive cases can be found in Table 1.

**Table 1:** Clinical characteristics of children less than 3 years of age evaluated for TB.

	TB cases^1^ (n = 43^#^)		Non-TB cases^2^ (n = 723^#^)	
	n	%	n	%
**Sex**				
* Male*	19	44.2	403	44.3
* Female*	24	55.8	320	55.7
**Age (months)**				
* < 12*	9	20.9	132	18.3
* 12–23*	16	37.2	373	51.6
* 24–35*	18	41.9	218	30.1
**Cough**				
* Yes*	8	18.6	144	19.9
* No*	35	81.4	579	80.1
**Fever**				
* Yes*	5	11.6	45	6.2
* No*	38	88.4	678	93.8
**Malnutrition**				
* Yes*	31	72.1	616	85.2
* No*	12	27.9	107	14.8
**HIV status**				
* Infected*	20	46.5	80	11.1
* Uninfected*	23	53.5	643	88.9
**TST result**				
* Positive*	23	54.8	51	7.1
* Negative*	19	45.2	671	92.9
**BCG scar**				
* Present*	42	97.7	720	99.6
* Absent*	1	2.3	3	0.4

Definitions: cough for ≥ 14 days not responding to appropriate course of antibiotics; referred fever 14 days after common causes like malaria or pneumonia were excluded; weight loss/failure to thrivedefined as under 60% weight for height, failure to gain weight for more than 2 months or any loss of weight not responding to nutritional intervention

^1^TB cases—includes confirmed and probable TB cases

^2^Non-TB cases—includes possible, unlikely and not TB as defined in the consensus case definitions for intra-thoracic TB in children by an expert panel.[10]

^#^ Missing values implies that the observation was not recorded.

Air space opacification was the most frequent parenchymal abnormality observed at admission, present in 18.5% (142/766) of all presumptive cases, followed by airway compression (or tracheal displacement), which was observed in 5.5% of all cases (45/766). Soft tissue density suggestive of lymphadenopathy was seen in 2.1% of all presumptive cases.

**Fig 1:**
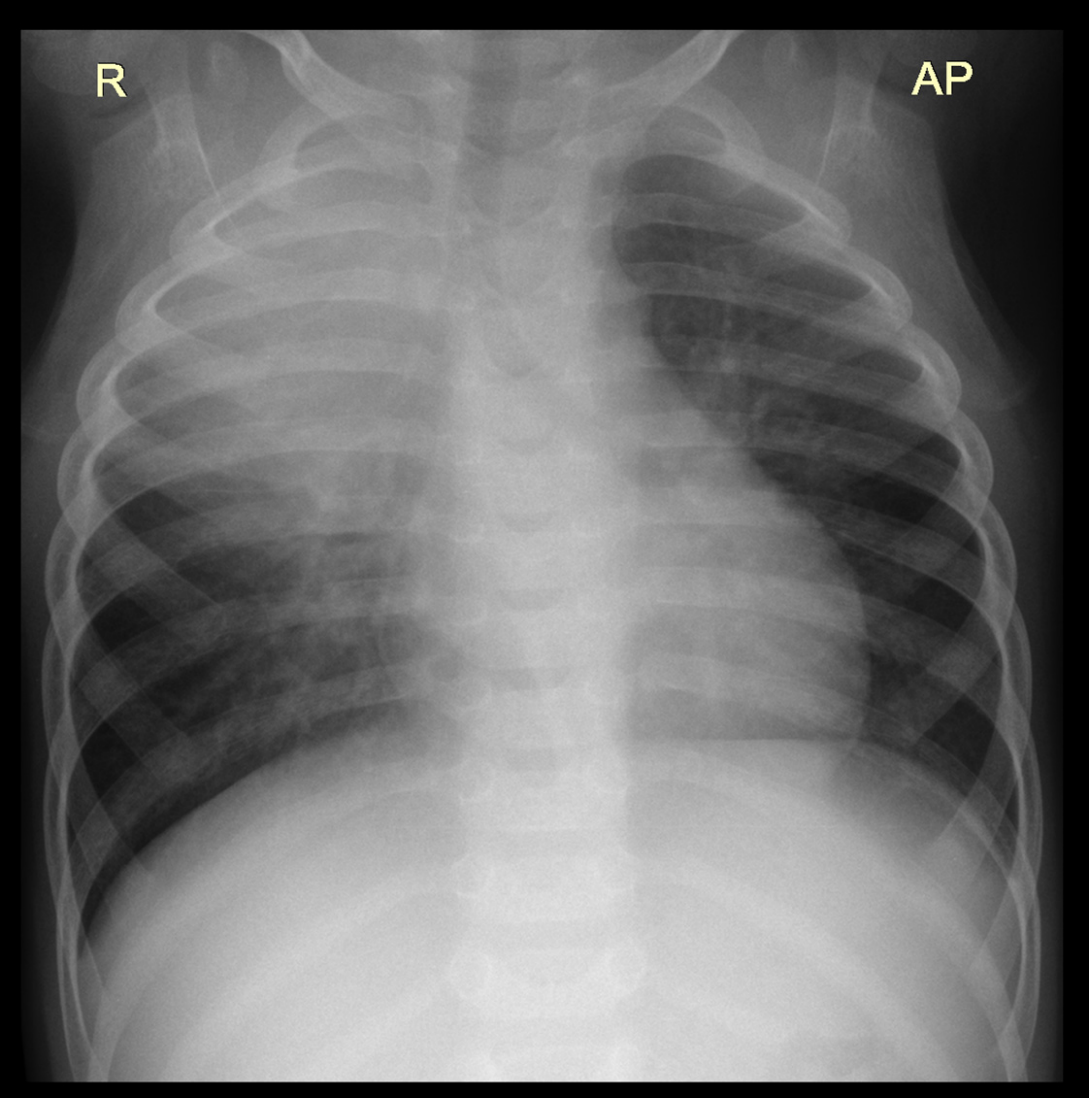
Air space opacification in the left upper lobe in a 20 month old male HIV-uninfected infant.

**Fig 2:**
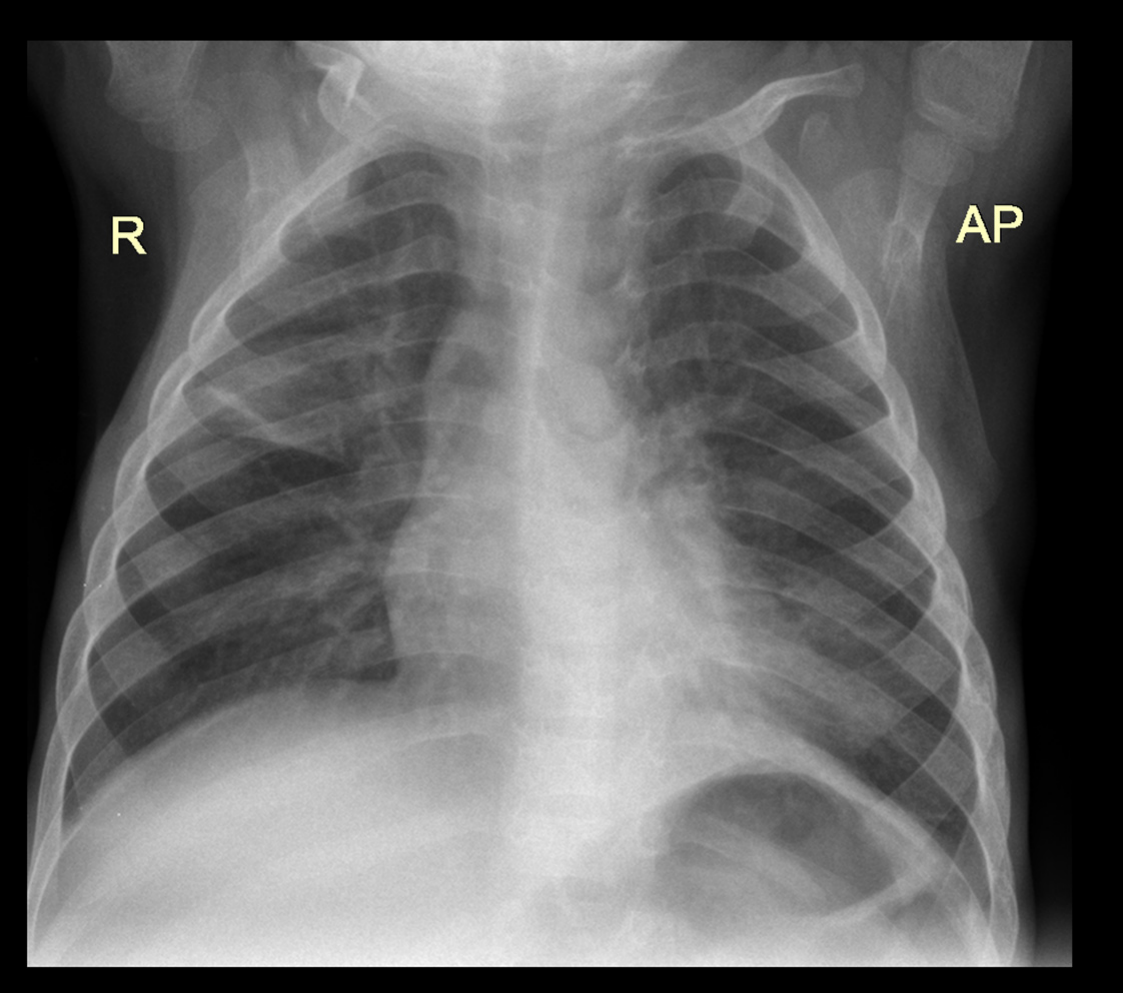
Bilateral bronchoneumonia with perihilial lymph node enlargement and possible left bronchius compression in an 5 month old male HIV infected TB case. AP view.

**Fig 3:**
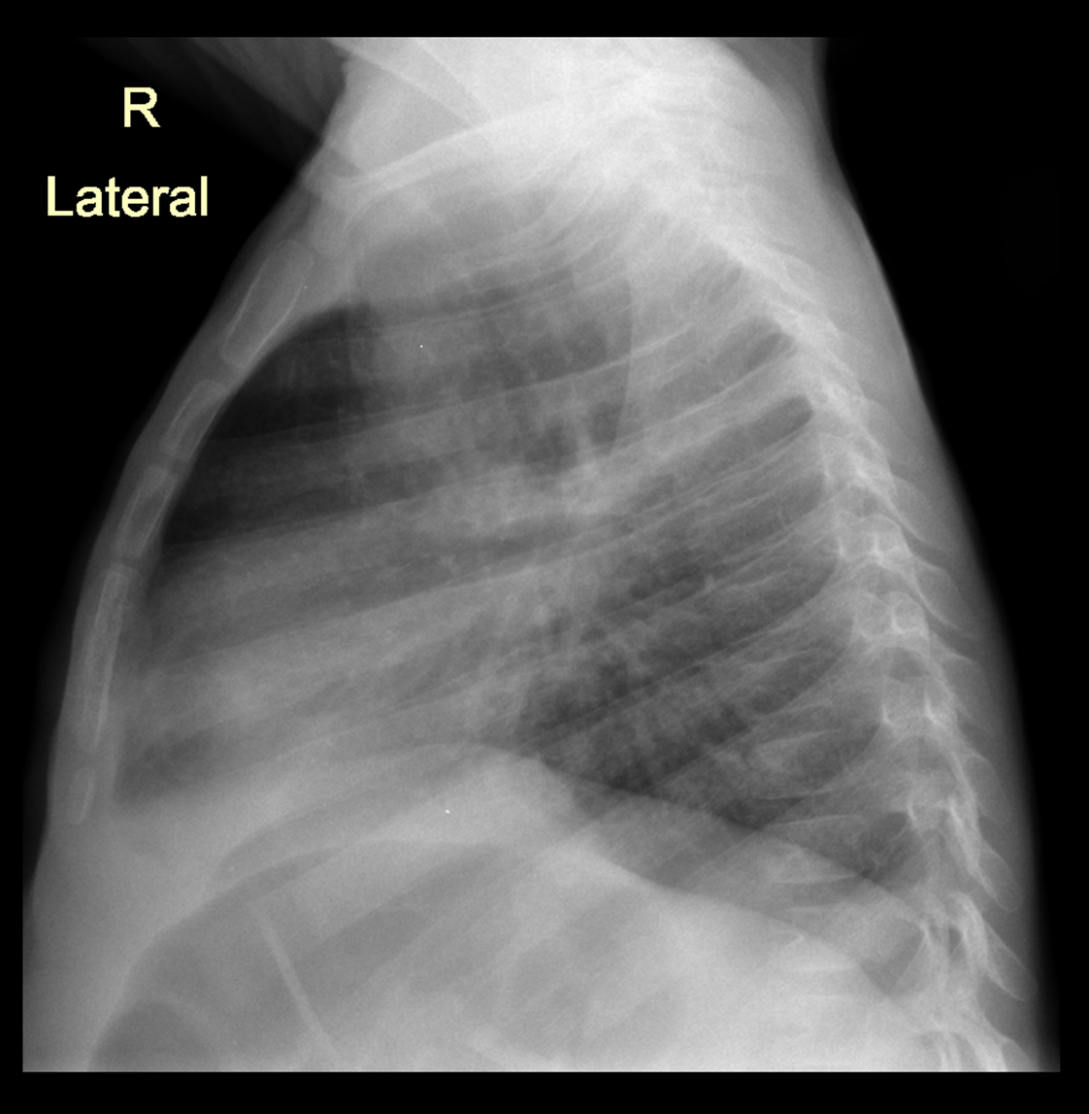
Bilateral bronchoneumonia with perihilial lymph node enlargement and possible left bronchius compression in an 5 month old male HIV infected TB case. Lateral View.

**Fig 4:**
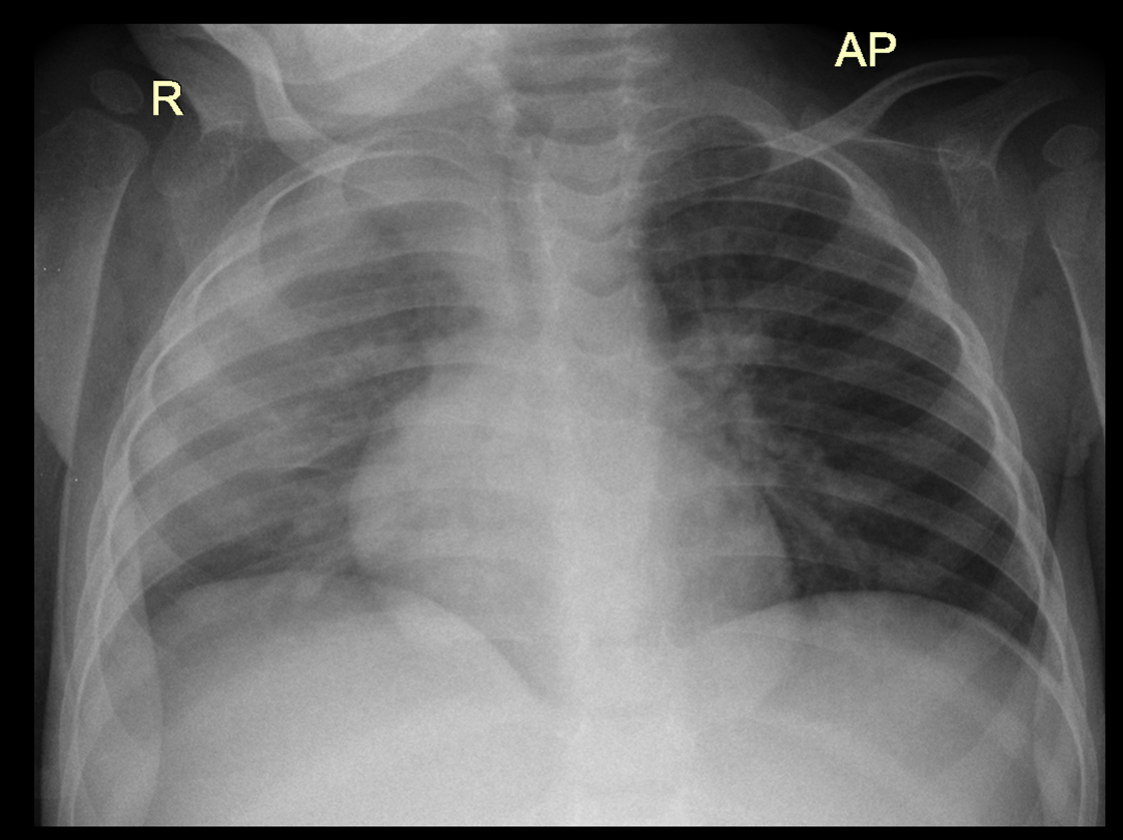
Right sided pleural effusion with no signs of primary disease in a 19 month old female HIV positive patient.

**Fig 5:**
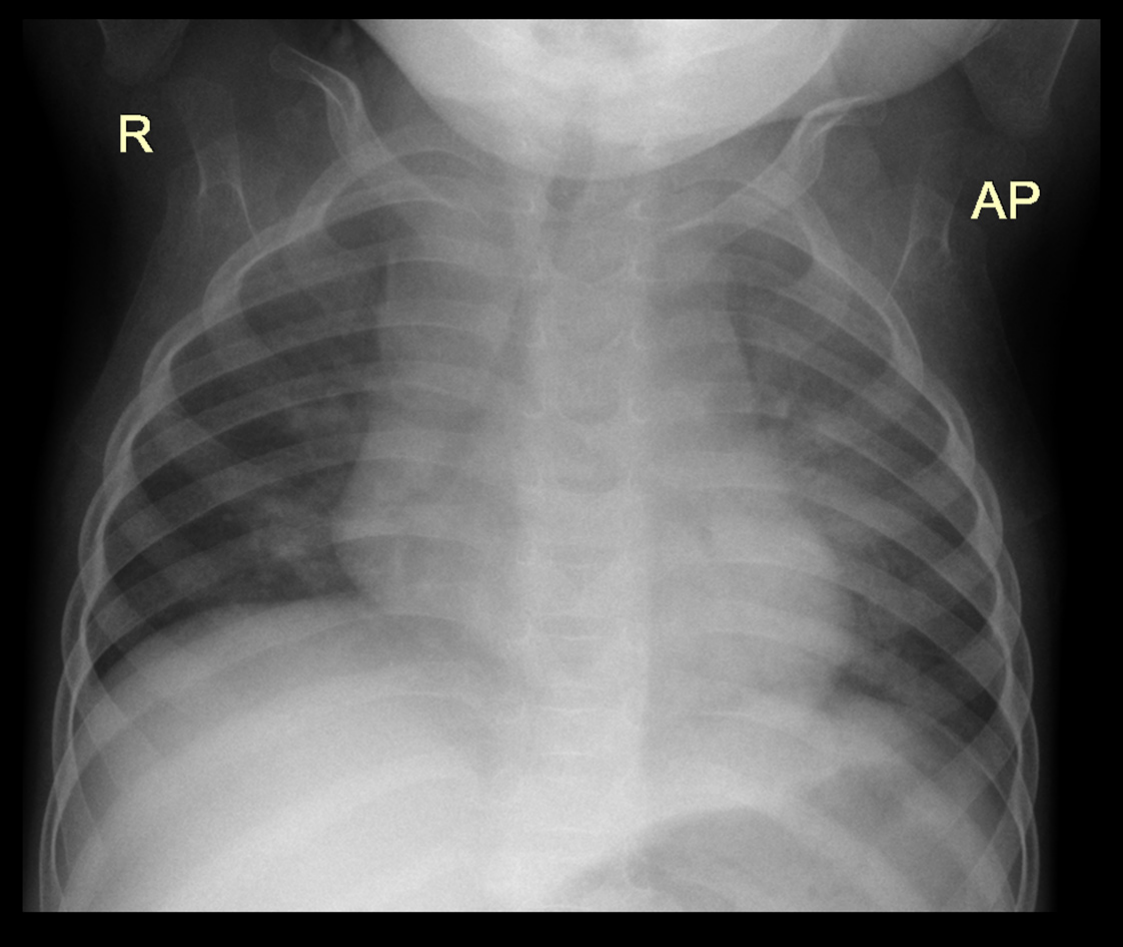
Airway compression and displacement of the left main bronchus with some consolidation in the left inferior lobe in a 17 month old HIV negative female.

The most frequent lesion found in TB probable and confirmed cases was air space opacification (65.1%, Fig 1) followed by soft tissue density suggestive of lymphadenopathy (17.1%, Figs 2 and 3), pleural effusion (7.0%,Fig 4) and airway compression (5% Fig 5). These findings were much more common in TB cases than in non TB cases: OR 8.9 (95% CI: 1.6–50.5), 17.2 (95% CI: 5.7–52.1) and 11.0 (95% CI: 2.5–48.6) respectively. Radiological characteristics of TB cases are described in Table 2.

**Table 2:** Chest X Ray characteristics according to TB disease status.

	All TB presumptive cases	TB cases^1^ (n = 43)		Non TB cases^2^ (n = 723)		OR (95% CI)	p valpue
		n	%	n	%		
**Age (months)**							
< 12	141	9	(20.9)	132	(18.3)	1.00	
12–23	389	16	(37.2)	373	(51.6)	1.59(0.69–3.68)	0.028
24–35	236	18	(41.9)	218	(30.2)	1.92(0.96–3.85)	0.06
**Airway compression or tracheal displacement**							
Yes	6	2	(4.8)	4	(0.6)	8.88 (1.56–50.48)	0.039
No	750	40	(95.2)	710	(99.4)	1.00	
**Soft tissue density suggestive of lymphadenopathy**							
Yes	15	7	(17.1)	8	(1.2)	17.17(5.66–52.06)	<0.001
No	701	34	(82.9)	667	(98.8)	1.00	
**Air space opacification**							
Yes	142	28	(65.1)	127	(17.8)	8.61 (4.36–17.03)	<0.001
No	614	15	(34.9)	586	(82.2)	1.00	
**Nodular Picture (miliary or larger widespread and bilateral)**							
Yes	1	1	(2.3)	0	(0.0)	—	
No	761	42	(97.7)	719	(100.0)	1.00	
**Pleural Effusion**							
Yes	8	3	(7.1)	5	(0.7)	11.02(2.50–48.57)	0.007
No	755	39	(92.9)	716	(99.3)	1.00	
**Other abnormalities (calcified parenchyma)**							
Yes	2	0	(0.0)	2	(0.3)	—	
No	760	42	(100.0)	718	(99.7)	1.00	

^1^TB cases—includes confirmed and probable TB cases

^2^non-TB cases—includes possible, unlikely and not TB as defined in the consensus case definitions for intra-thoracic TB in children by an expert panel.[10]

When cases for all variables do not add up, it means that the observation was not visible in the radiography.

* P value obtained through *χ* ^2^ (chi-squared) test or Fisher’s exact test (when applicable).

**Fig 6:**
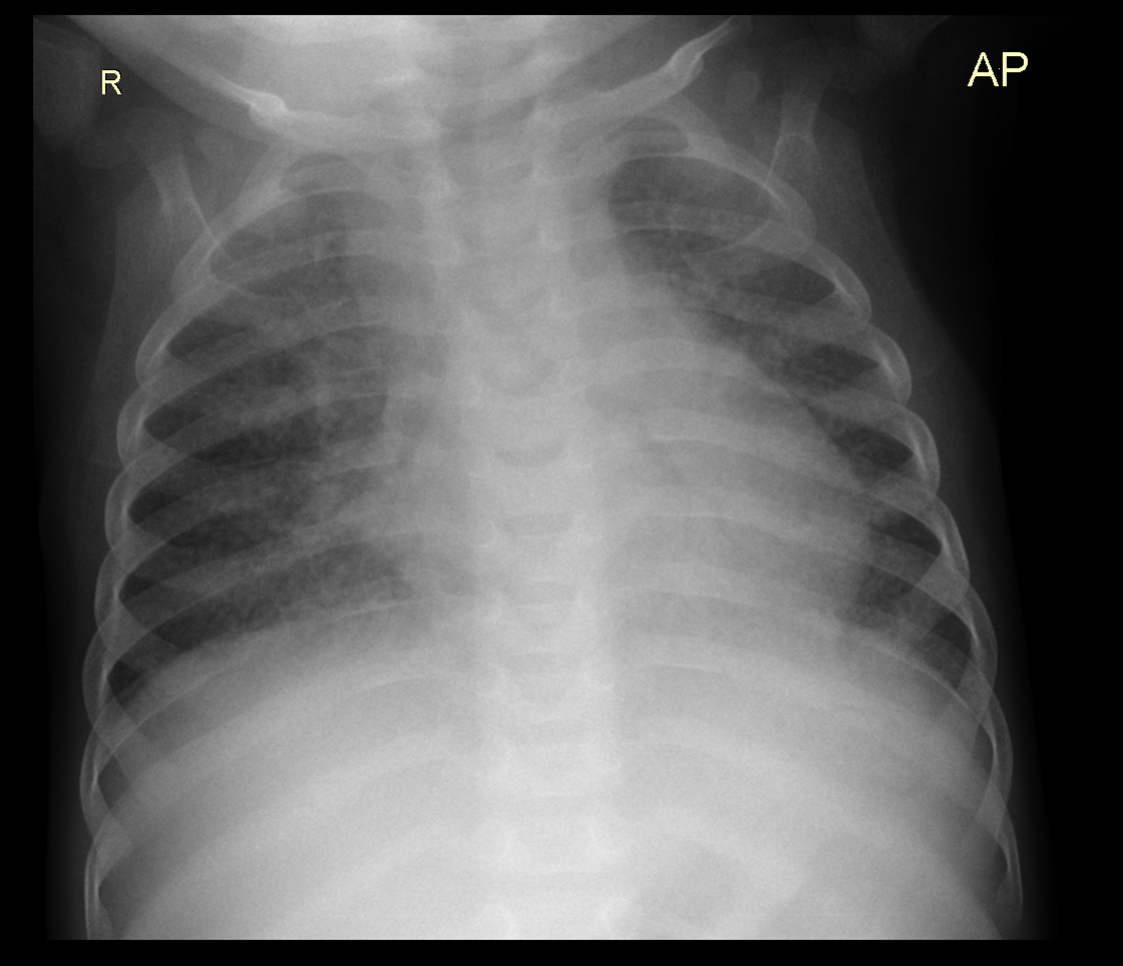
Millet sized nodules of a miliary tuberculosis in an 13 month old HIV infected male.

All 3 cases pleural effusion cases, as well as the only miliary case (Fig 6) occurred in HIV positive cases. Soft tissue suggestive of lymphadenopathy was more frequently found in HIV positive TB cases than in HIV negative (21.1% vs 13.6% respectively), although the association of this finding to HIV status was not statistically significant.

Among TB cases, the most frequent location of soft tissue density suggestive of lymphadenopathy was the mediastinal region of the upper right lobe, followed by mediastinal portion of the middle right lobe and anterior paratracheal region (as seen in the lateral view). The two TB cases with air compression signs affected the right bronchus. Air space opacification was more frequent in middle right lobe, followed by lower right and lower left lobe. Out of the three TB cases presenting pleural effusion, two of them were observed in the right lobe.

Out of the 43 TB cases, only 20 had a chest X ray during the last month of treatment or the next 4 months after treatment was finished. Air space opacification, present in 65% of children at diagnosis was still present in 50% of cases with available follow up CXR, with no difference depending on HIV status. Lymphadenopathy which was observed in 7 cases at diagnosis, was still present in 2 cases at follow up. Of the three cases with pleural effusions at diagnosis, two of them had follow up CXR and in one of them, the effusion persisted at 5 month follow up (Table 3).

**Table 3:** Chest X-ray characteristics, before and after TB treatment, in child TB cases less than 3 years of age.

		On Admission		5–10 month after treatment initiation	
		HIV infected	HIV uninfected	HIV infected	HIV uninfected
	**Number of cases with Chest X Ray**	20	23	11	9
**Radiographic findings**	**Airway compression or tracheal displacement**	1	1	0	0
	**Soft tissue density suggestive of lymphadenopathy**	4	3	1	1
	**Air Space Opacification**	13	15	6	4
	**Nodular/Miliary Picture**	1	0	0	0
	**Pleural Effusion**	3	0	1	0
**Abnormal X ray**		16	16	7	4

* Total number of cases by HIV status.

## Discussion

To our knowledge this is one of the few studies from a high TB burden country reporting in a systematic manner the results of digital CXR from young TB presumptive infants, following most recommendations from the Expert Panel for evaluation of TB diagnostic tools in children.[10] Although the CXR review tool developed by S. Andronikou and the South African Tuberculosis Vaccine Initiative (SATVI) has already been used for TB case classification purposes in at least one large vaccine clinical trial [19], this is the first report providing detailed results using this standardized template.

In our study, the most frequent lesion found in both TB cases and presumptive cases in was air space opacification, although the odds for of presenting lymphadenopathy among TB cases compared to non TB cases, was the highest in comparison to the other lesions. Thus, this finding goes in line with the general expert opinion that states that mediastinal/hilar lymphadenopathy is the radiological hallmark in childhood tuberculosis [12,20,21]. However, given that the observation of lymphadenopathy is more frequent in younger children compared to adolescents, as reported by other authors [11,21,22], our results clearly show a lower prevalence of this typical finding among TB cases. This could be due to various reasons. Parenchymal lesions such as air space opacification (whether due to TB or other concomitant pathologies), could hinder the visualization of enlarged lymph nodes, although both findings could be present at the same time. This could be enlightened by the performance of US or CT scan [23]. It could also happen that the high level of malnutrition among our TB cases (72%) and the presence of other immune deficiencies (HIV infection), which are known factors affecting disease progression, might have favored the bronchopneumonic consolidation, as an evolved lesion from regional lymph node disease. Nonetheless, the fact that parenquimal consolidation was the most frequent radiological finding among TB cases complicates the radiological distinction from common bacterial pneumonia, hindering further TB diagnosis.

The analysis of RX patterns depending on HIV status did not show any relevant difference on radiological findings. Although there is no literature comparing Rx findings depending on HIV status for this age group, since 46% of our TB cases were HIV positive, we expected to have more atypical presentations of TB for this group, with more cases of miliary TB and massive mediastinal glands. Clearly, the CXR characterization on HIV positive infants with tuberculosis, which depends on their immune status (CD4 counts), presence of reconstitution syndrome or HAART treatment, needs to be further assessed with larger studies

Although by the end of treatment it was expected that 66% of abnormal chest radiographs at diagnoses would have disappeared [24], we found a lower proportion of disease free compatible CXR (50%). In our study, in 50% of children with available follow up, consolidation persisted, although with different characteristics and sometimes locations. It could be that disease resolution is slower in a population with high level of undernutrition and HIV or some consolidations might be explained by a paradoxical reaction due to immune reconstitution. Another explanation might be that some of the cases were misdiagnosed as TB when other causal pathogens could have caused the pathological image (ie, pneumonias).

This study had several methodological limitations. First, although most patients had an AP projection, some of them lacked the lateral projection, thus, given the pivotal role of lateral views for lymphadenopathy evaluation, some of them could have been missed. Second, due to the limited number of TB cases identified in these studies, the conclusions, especially on the association of HIV status to different radiological findings among TB cases, are not robust, and studies with greater sample sizes are needed. Second, although we have not observed any cavities, calcified parenchyma (Ghon focus) or vertebral spondylitis among TB cases, we have observed that the template does not allow differentiating among these lesions, and reporting of the location might not be possible. We recommend that these lesions, which have been found in other CXR evaluations, could be reported separately. Third, although it has been recommended that the readings from the two reviewers were masked to clinical data and discrepancies resolved by a third reader, this was not possible due to logistical and personnel constraints (only one was masked). Poor inter observer agreement among reviewers regarding lymphadenopathy evaluation in children has been reported, so this limitation, together with the inadequate capturing of differences in opinion, could have reduced the accuracy of the findings which are being reported.

Chest X ray evaluation remains a crucial tool for TB diagnosis in childhood due to the difficulty of isolating TB from sputum or other human samples and the unavailability of reliable TB diagnostic methods in this age group. The correct interpretation of CXR for diagnostic purposes in both clinical practice and research makes standardization of reporting critical. There are some scoring systems for adults which are especially useful to discard TB disease [25,26], but this type of scoring systems are inexistent for children, which have distinct radiological manifestations and where HIV infection, severe malnutrition adds another level of complexity given the absence of atypical presentation of radiological manifestations. Thus, there is a need for improved scoring systems for pediatric populations.

## Conclusions

Hilar lymph node enlargement, often regarded as the typical radiological feature of TB in children, was seen in a minority of TB cases. Parenchymal consolidation was the most common finding, complicating radiological distinction from common bacterial pneumonia in children less than 3 years of age. These findings underscore the importance of not ruling out TB despite the absence of the most characteristic radiological findings, and the need of combining radiological information with other signs, symptoms or epidemiological information in cases where laboratory confirmation is not possible. The role of HIV infection and malnutrition in the radiological presentation of TB among young children, highly present in this rural population of Southern Mozambique, deserves to be further studied.
