# Correction: Radiological Findings in Young Children Investigated for Tuberculosis in Mozambique

**DOI:** 10.1371/journal.pone.0133338

**Published:** 2015-07-15

**Authors:** Alberto L. García-Basteiro, Elisa López-Varela, Orvalho Joaquim Augusto, Kizito Gondo, José Muñoz, Jahit Sacarlal, Ben Marais, Pedro L. Alonso, José L. Ribó

There are errors in the Author Contributions. The correct contributions are: Conceived and designed the experiments: PLA JS JM. Collected data: ELV JLR KG JS. Interpreted images: ELV JLR. Analyzed the data: ALGB ELV OJA. Wrote the paper: ALGB ELV BM.
